# Post-eruptive flooding of Santorini caldera and implications for tsunami generation

**DOI:** 10.1038/ncomms13332

**Published:** 2016-11-08

**Authors:** P. Nomikou, T. H. Druitt, C. Hübscher, T. A. Mather, M. Paulatto, L. M. Kalnins, K. Kelfoun, D. Papanikolaou, K. Bejelou, D. Lampridou, D. M. Pyle, S. Carey, A. B. Watts, B. Weiß, M. M. Parks

**Affiliations:** 1 National and Kapodistrian University of Athens, Department of Geology and Geoenvironment, Panepistimioupoli Zografou, Athens 15784, Greece; 2 Laboratoire Magmas et Volcans, Université Blaise Pascal—CNRS—IRD, Campus des Cézeaux, Aubière 63178, France; 3 Institute for Geophysics, University of Hamburg, Bundesstrasse 55, Hamburg 20146, Germany; 4 Department of Earth Sciences, University of Oxford, South Parks Road, Oxford OX1 3AN, UK; 5 Géoazur CNRS, Bât 4, 250 rue Albert Einstein, Sophia Antipolis, Valbonne 06560, France; 6 Department of Earth Sciences, Durham University, Durham DH1 3LE, UK; 7 Graduate School of Oceanography, University of Rhode Island, 215 S. Ferry Road, Narragansett, Narragansett, Rhode Island 02882, USA; 8 Nordic Volcanological Center, Institute of Earth Sciences, University of Iceland, Reykjavík IS-101, Iceland

## Abstract

Caldera-forming eruptions of island volcanoes generate tsunamis by the interaction of different eruptive phenomena with the sea. Such tsunamis are a major hazard, but forward models of their impacts are limited by poor understanding of source mechanisms. The caldera-forming eruption of Santorini in the Late Bronze Age is known to have been tsunamigenic, and caldera collapse has been proposed as a mechanism. Here, we present bathymetric and seismic evidence showing that the caldera was not open to the sea during the main phase of the eruption, but was flooded once the eruption had finished. Inflow of water and associated landsliding cut a deep, 2.0–2.5 km^3^, submarine channel, thus filling the caldera in less than a couple of days. If, as at most such volcanoes, caldera collapse occurred syn-eruptively, then it cannot have generated tsunamis. Entry of pyroclastic flows into the sea, combined with slumping of submarine pyroclastic accumulations, were the main mechanisms of tsunami production.

## Abstract (web summary)

The Bronze Age eruption of Santorini is known to have generated tsunamis with caldera collapse as the likely mechanism. However, new bathymetric and seismic data presented by Nomikou *et al*. show that the entry of pyroclastic flows into the sea is the most likely tsunami-generating mechanism at Santorini.

## Graphical abstract

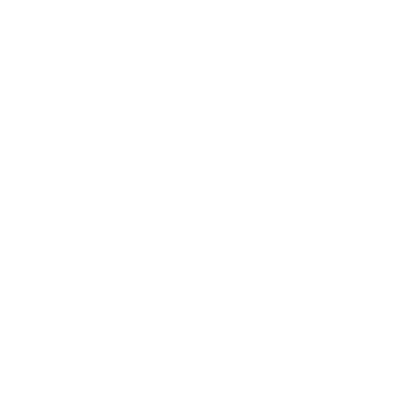

The Late Bronze Age (LBA) eruption was one of the largest of the Holocene period worldwide, discharging 30–80 km^3^ DRE (dense-rock equivalent) of silicic pyroclastic deposits, and triggering caldera collapse^1^,^2^,^3^,^4^,^5^. Repeated effusive eruptions since the LBA eruption have built up the Kameni Volcano within the resulting caldera^6^. The LBA caldera is 10 × 7 km wide, comprises three flat-floored basins around the Kameni edifices, and is connected to the sea by three straits (one to the NW and two to the SW; Fig. 1a)^7^,^8^,^9^. Recent seismic reflection profile studies have revealed three main stratigraphic units within the upper ∼200 m of the intracaldera fill, numbered 1 to 3 from the top-down^10^: flat-lying sediments from modern mass wasting of the caldera cliffs (unit 1), volcaniclastic sediments produced during the early submarine stages of Kameni Volcano (unit 2), and downfaulted material interpreted as the top of the LBA eruption products (unit 3). Other subdivisions of these same layers have been published^11^.

Our new constraints on the mechanisms of tsunami generation associated with the LBA eruption arise from a multi-beam bathymetric study, supplemented by seismic profiling, of the three straits connecting the caldera to the sea. Combined with information from onshore studies of the LBA eruption products^1^,^2^,^3^,^4^,^5^, the data allow us to show that the NW strait was carved by inrushing of the sea into the newly collapsed caldera once the eruption was over. This therefore rules out caldera collapse as a major mechanism of tsunami generation associated with the LBA eruption. We also show by modelling that the main phase of caldera flooding cannot have taken more than 2 days to arrive at completion.

## Results

### Structure of the NW and SW straits

**Figure 1:**
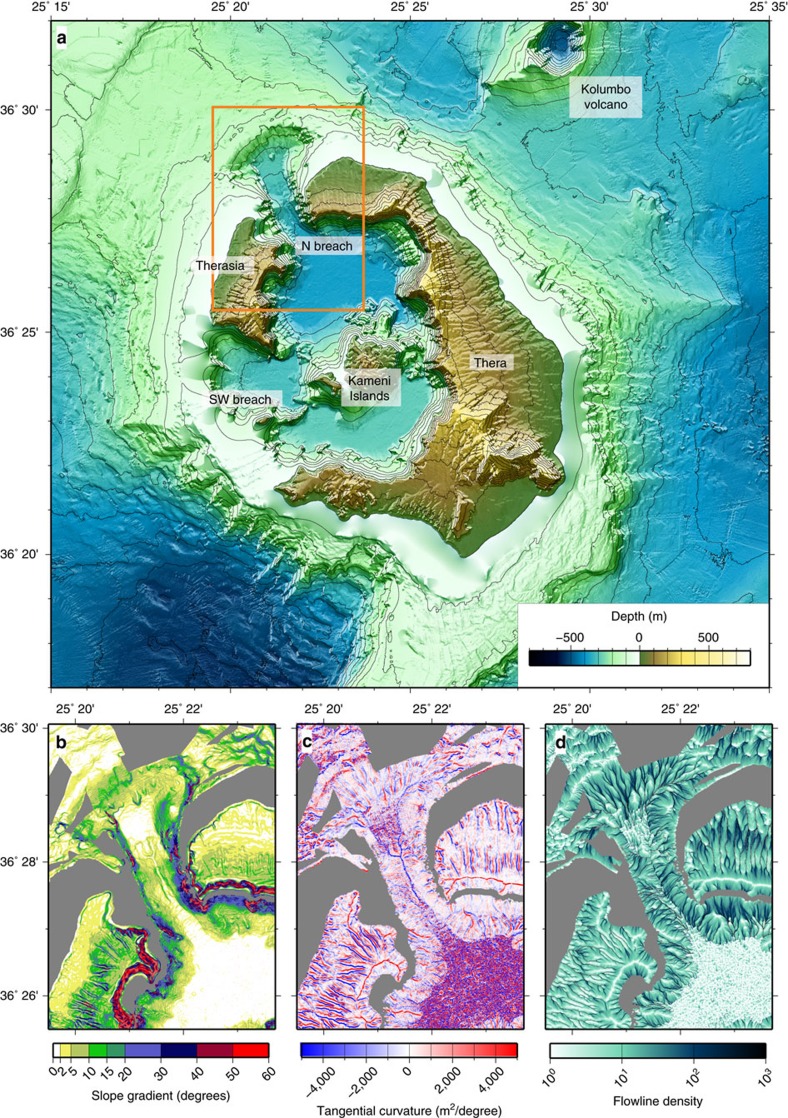
Topographic features of the Santorini onshore-offshore volcanic field. (**a**) Combined topographic map of Santorini Volcano based on onshore and offshore data. Orange box outlines the area of the northwest strait, shown as (**b**) a slope gradient map, (**c**) a tangential curvature map and (**d**) a flowline density map.

**Figure 2:**
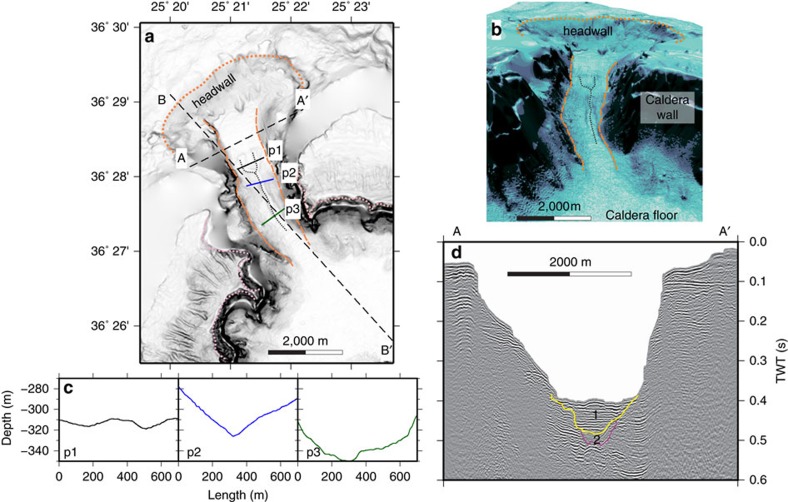
More detailed topography and seismic profiling of the NW strait of Santorini caldera. (**a**) Shaded slope gradient with the location of profiles shown in 2c (p1, p2 and p3), 2d (seismic profile A-A′) and Fig. 3 (seismic profile B-B′) marked. The dotted orange line marks the location of the erosional headwall, the dashed orange line the channel sides, the dotted pink line the rim of the caldera, and the dotted black line the small-scale drainages superimposed on the large-scale channel. (**b**) Perspective view of the NW strait. (**c**) Bathymetric cross sections perpendicular to the NW strait. (**d**) Seismic profile perpendicular to the strait axis, showing caldera-fill stratigraphic units 1 and 2 of ref. 10.

**Figure 3:**
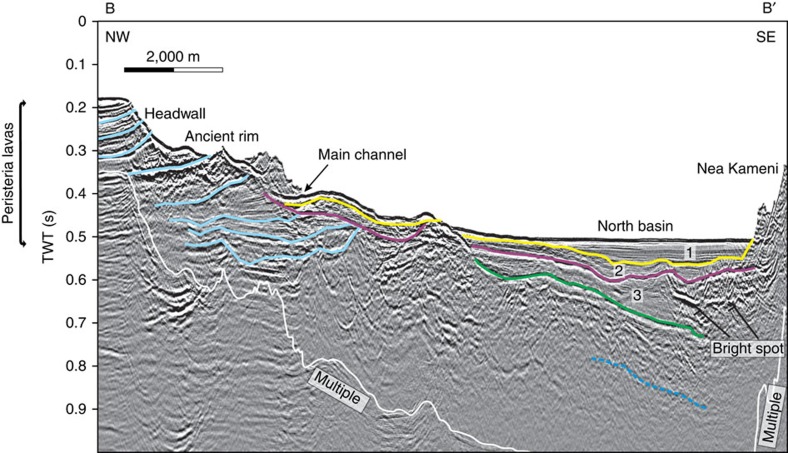
Interpreted multi-channel reflection seismic profile along the axis of the NW strait. Transect B-B′ in Fig. 2. Stratigraphic units 1–3 of ref. 10 are shown numbered. The light blue lines indicate stratigraphic horizons within Peristeria lavas, and the dotted dark blue line indicates the presence of a deep, but poorly defined reflector. What appears as an edifice in the upper part of the slope is a ledge of the channel rim. A TWT (two-way travel time) interval of 0.1 s corresponds approximately to a water depth interval of 75 m or a sediment interval of 100 m. Please refer to Supplementary Fig. 1 for the uninterpreted seismic profile.

The new multi-beam bathymetric data show that the NW strait consists of a 3 km long, 1 km wide, U-shaped, submarine channel with an arcuate headwall (Fig. 1a). The erosional morphology of the headwall, 2 km across and with slopes of 5–10°, is scarred by landslides and multiple narrow submarine drainages that converge towards the caldera (Figs 1b–d and 2a,b). In the middle course of the strait, two distinct drainages merge downstream to form a single V-shaped drainage (section p2 in Fig. 2c). A seismic reflection profile along the axis of the strait (Fig. 2a; Supplementary Fig. 1) reveals that the headwall is carved into NW-dipping, coherent lithologies representing the lava succession that comprises much of northern Santorini^12^. Prominent, more or less continuous, reflections are probably lavas and acoustically transparent layers are either tuffs^11^ or hemipelagic sediments^13^. Phase-reversed bright spots may indicate bedding-parallel fluid flow^11^. The NW-dipping reflectors underlie much of the strait, covered only by thin sediment layers of units 1 and 2 (ref. 10. To the SE, a seismic profile into the caldera shows all three sediment units (Fig. 3; ref. 11). A seismic reflection profile perpendicular to the strait axis (Fig. 2a,d) reveals superficial landslides on the margins and thin layers of units 1 and 2 on the floor (Fig. 2d). A prominent landslide deposit at the foot of the headwall can be distinguished (Fig. 1a,d). Deep faulting is not observed, beneath either the breach headwall or beneath the margins (Figs 2d and 3).

**Figure 4:**
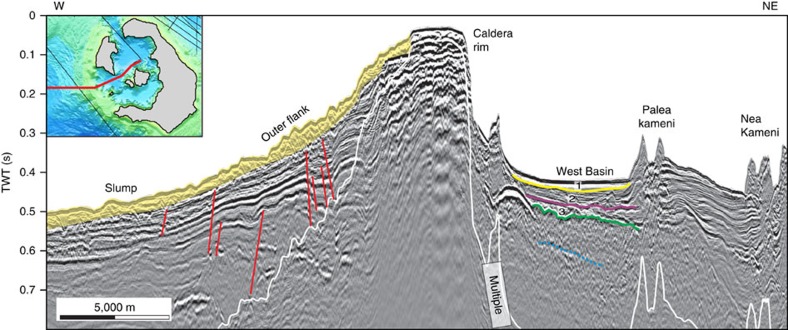
Interpreted W-NE striking multi-channel reflection seismic profile across the SW strait. This profile cuts across the outer edge of the western basin of Santorini caldera, as shown in the inset. Stratigraphic units 1–3 of ref. 10 are shown numbered; The dotted dark blue line indicates a deep, but poorly defined reflector; faults are shown in red. A TWT (two-way travel time) interval of 0.1 s corresponds approximately to a water depth interval of 75 m or a sediment interval of 100 m. Please refer to Supplementary Fig. 2 for the uninterpreted seismic profile.

**Figure 5:**
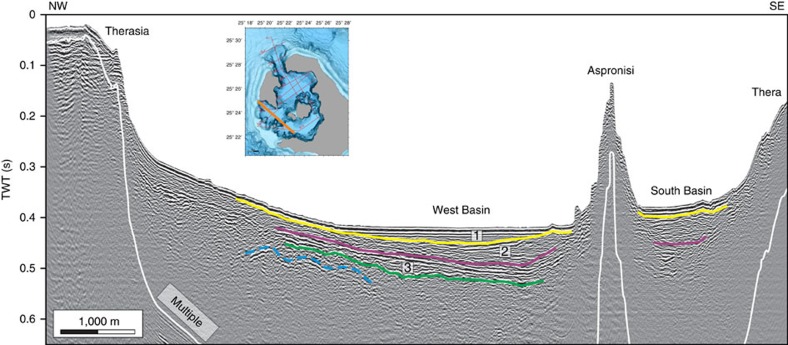
Interpreted NW-SE sparker reflection seismic profile. This profile crosses the two SW straits, as shown by the orange line in the bathymetric map inset. Stratigraphic units 1–3 of ref. 10 are shown numbered; The dotted dark blue line indicates the presence of a deep, but poorly defined reflector. A TWT (two-way travel time) interval of 0.1 s corresponds approximately to a water depth interval of 75 m or a sediment interval of 100 m. Please refer to Supplementary Fig. 3 for the uninterpreted seismic profile.

The southwest straits are morphologically fresh, and have landslide scars with well-preserved headwalls and intervening septa (Fig. 1a). The headwalls are steeper than that of the NW strait, and are less scarred by secondary slumping and drainage channels. Seismic reflection profiles of the SW straits reveal the fill of the western basin (Figs 4 and 5, Supplementary Figs 2 and 3). High-resolution bathymetry of the SW straits (Supplementary Fig. 4) is presented for comparison with that for the NW strait.

### Origin of the straits

We interpret the NW strait as a flood-modified landslide scar, formed by northward propagating regressive erosion and landsliding, and the headwall as a huge fossilized water chute (Figs 1a and 2a,b). It is clearly erosional in origin, since no deep faulting is observed. The arcuate headwall scar is concave towards the caldera, thus implying sliding and water flow into the caldera. The large-scale sea floor morphology, with its wide, rounded headwall scar and narrow, steep-sided passage, shows marked similarities to features commonly observed when a manmade dam fails with a sudden outflow of water (as shown on Figs 1, 5 and 6 from ref. 14, on front cover and Plates 11.1, 11.5, 11.6 from ref. 15, in figures 3 and 5 from ref. 16). This strongly suggests a sudden breach of the caldera wall analogous to a dam failure. The breach must have required significant force, given the structural integrity of the channel walls. A strong inward-directed flow of water suggests that the caldera was either dry, or that the level of water was much lower than that of the surrounding sea, when the breach took place. The onset of inflow by the sea was probably accompanied by large-scale landsliding into the caldera, followed by erosion due to the rapidly flowing water. The much smaller-scale drainage pattern superimposed on the strait sea floor morphology (Fig. 1c,d) either formed at a late stage of the flooding event, or was cut by the present-day flow (8–19 cm s^−1^) of cold Mediterranean bottom waters into the warmer waters of the caldera^17^.

In contrast, the morphological freshness of the two SW straits (Supplementary Fig. 4) suggests that they formed by slumping once the caldera was already flooded (Fig. 1a). We envisage that, during formation of the NW strait, there was little water inside the LBA caldera (high pressure difference with outside the caldera), whereas during formation of the SW straits the caldera was already flooded (small pressure difference with outside the caldera).

### Constraints on the onset of caldera flooding

Constraints on the timing of the caldera flooding event are provided by previous studies of the LBA eruption products, and of studies of the changes in morphology of the volcano resulting from the eruption and associated caldera collapse. The eruption took place in four main phases. It began with a Plinian phase (phase 1) from a subaerial vent, then became phreatomagmatic (phases 2 and 3) (refs 1, 2, 3, 4, 5). The main phase of the eruption (phase 4) involved outpouring of hot, fluidized pyroclastic flows, forming multiple ignimbrite (deposit from a pyroclastic flow) fans^1^,^2^,^3^,^5^.

**Figure 6:**
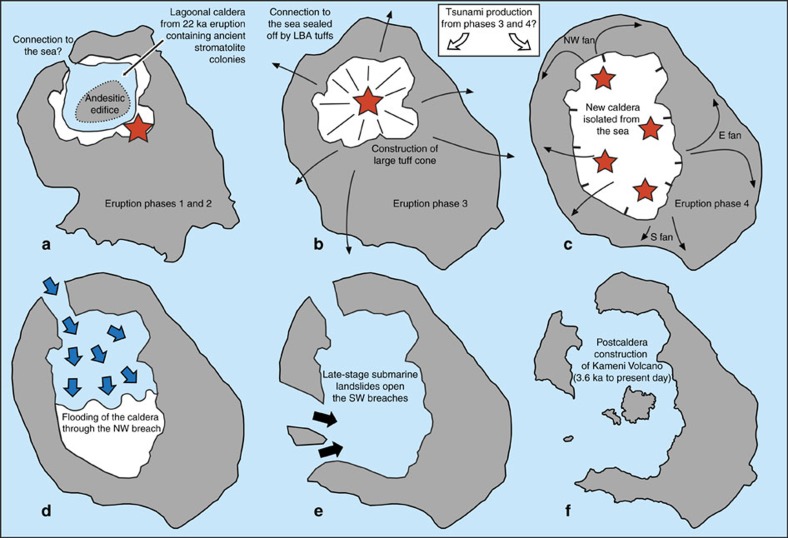
Summary of the development of Santorini caldera before and during and following the LBA eruption. (**a**–**c**) Pre-eruptive morphology of the volcano^18^,^19^,^20^,^21^ and the main phases of the eruption^1^,^2^,^3^,^4^,^5^. Before the eruption there existed an ancient caldera in the northern half of the volcanic field^18^,^19^. This caldera was lagoonal, as shown by the presence of fragments of ancient travertine, stromatolites and brackish to marine fauna in the LBA ejecta^20^,^21^. There was also an andesitic edifice within this caldera^5^. In eruptive phase 1 a Plinian eruption took place, which in phase 2 was joined by the production of syn-plinian pyroclastic surges. In phase 3, eruption of ‘cold' phreatomagmatic pyroclastic flows constructed a large tuff cone that filled the old caldera, cutting it off from the sea. In phase 4, eruption of hot pyroclastic flows took place from multiple subaerial vents, forming at least three ignimbrite fans (NW, E and S), and associated caldera collapse enlarged and deepened the ancient caldera. The main eruptive vents are shown in these figures as red stars (locations well constrained for phases 1 to 3, but speculative for phase 4). Black arrows show schematic emplacement vectors for the pyroclastic flows of phases 3 and 4. (**d**,**e**) Post-eruptive opening of the NW and SW straits (based on the present research). At the end of the eruption the caldera was dry and isolated from the sea, probably due to thick accumulations of LBA tuff. The sea first broke through to the NW, where a combination of water erosion and landslip carved out the NW strait and flooded the caldera (blue arrows in **d**) in less than a couple of days. Submarine landslides (black arrows in **e**) then opened up the SW straits once the caldera was largely flooded. (**f**) The present-day volcanic field.

Before the eruption, Santorini already had a shallow caldera that had formed during an eruption 18 ky previously^5^,^18^,^19^ (Fig. 6a). This ancient caldera was lagoonal, as inferred from fragments of travertine, stromatolites and brackish to marine fauna in the LBA ejecta^20^,^21^. The eruptive vent was situated outside the caldera lagoon during phase 1 of the LBA eruption, then migrated into it during phases 2 and 3, causing phreatomagmatic explosions^3^,^5^ (Fig. 6b). Phase 3, the most violent phreatomagmatic phase, used up most of the water in the lagoon, and built up a huge tuff cone that probably cut off any connection to the sea^4^. The subsequent eruption of hot, fluidized pyroclastic flows during phase 4 shows that, by the end of phase 3, the caldera was dry and the vents were subaerial^3^,^5^. Caldera collapse triggered by the LBA eruption deepened and widened the old caldera^3^,^4^,^5^. The pyroclastic flow deposits of phase 4 are rich in rock debris of diverse lithologies, suggesting that collapse took place mostly during phase 4 (refs 1, 3, 5) (Fig. 6c). Indeed it is known that caldera collapse takes place syn-eruptively at many calderas worldwide, although final settling may continue after the eruption^22^,^23^,^24^.

The newly collapsed LBA caldera must have been essentially dry by the end of the eruption, and isolated from the sea by thick accumulations of LBA tuff, because there was no return to phreatomagmatic activity following the hot pyroclastic flows of phase 4 (ref. 3). Moreover, phase 4 ignimbrite bordering each side of the NW strait is known from lithic thermal remnant magnetism analysis to have been emplaced hot (150–350°C) (ref. 25), and it contains none of the phreatomagmatic ashes typically produced when hot pyroclastic flows enter the sea. Hence, by the end of phase 3, the region between present-day Thera and Therasia islands must have been above sea level, probably due to a thick accumulation of phase-3 tuffs, with this barrier remaining intact throughout phase 4 until the end of the eruption (Fig. 6c). Then, once the caldera had largely collapsed, this wall of easily eroded tuffs failed, allowing the sea to rush in, accelerated by retrogressive landsliding into the caldera (Fig. 6d). That this occurred once the eruption was over would explain the apparent lack of LBA products on the floor of the NW strait (Figs 2d and 3). Finally, the two remaining SW straits collapsed once the caldera was already largely flooded, accounting for their fresh landslide morphologies (Fig. 6e).

Debris from these breaching events has not been recognized on seismic profiles inside the caldera (Figs 2d and 3). However, the high energy of water flow associated with the NW breach may have fragmented the debris and scattered it across a large area inside the caldera. We have estimated the total volume of rock removed to create the NW strait as 2.0–2.5 km^3^ (Supplementary Figs 5 and 6). The joint volume of seismic units 1, 2 and 3, in the northern basin, is only 0.94–1.1 km^3^ (as shown in figures 9–11 from ref. 10). The volume of material removed during the breach therefore cannot be accommodated in these units. Although unit 3 has been interpreted previously as the top of the LBA tuff succession^10^, we speculate that it might be composed of sediment deposited during the waning phase of the flood event, such as mass flows and suspension fallout of sediment from the highly turbid water column. The main layer of landslide and flood debris from the NW breach would then lie, unresolved, beneath unit 3, and the LBA intracaldera tuffs below that.

### Dynamics and duration of flooding

Initially, the flooding event through the NW strait would have been analogous to those associated with dam failure, with rapid erosion of the retaining wall leading to inflow. However, lake- or dam-breach floods cease once the upper reservoir is empty. In the LBA case the upper reservoir (the sea) was effectively infinite, and the flood stopped when the lower reservoir (the caldera) filled up to sea level. Thus the late-stage dynamics was different: at the end of a dam failure, the water still flows down a similar topographic drop, giving it similar potential energy drop per unit volume of water, whereas in our case, the potential energy drop decreases as the caldera fills. The erosive power of the influx will also decrease as it flows into increasingly deep water. A close, if smaller-scale, analogue was the flooding of a Malaysian open cast tin mine, when the wall separating the mine from the sea collapsed^26^. A much larger-scale analogue was the flooding of the Black Sea 8,400 years BP^27^.

**Figure 7:**
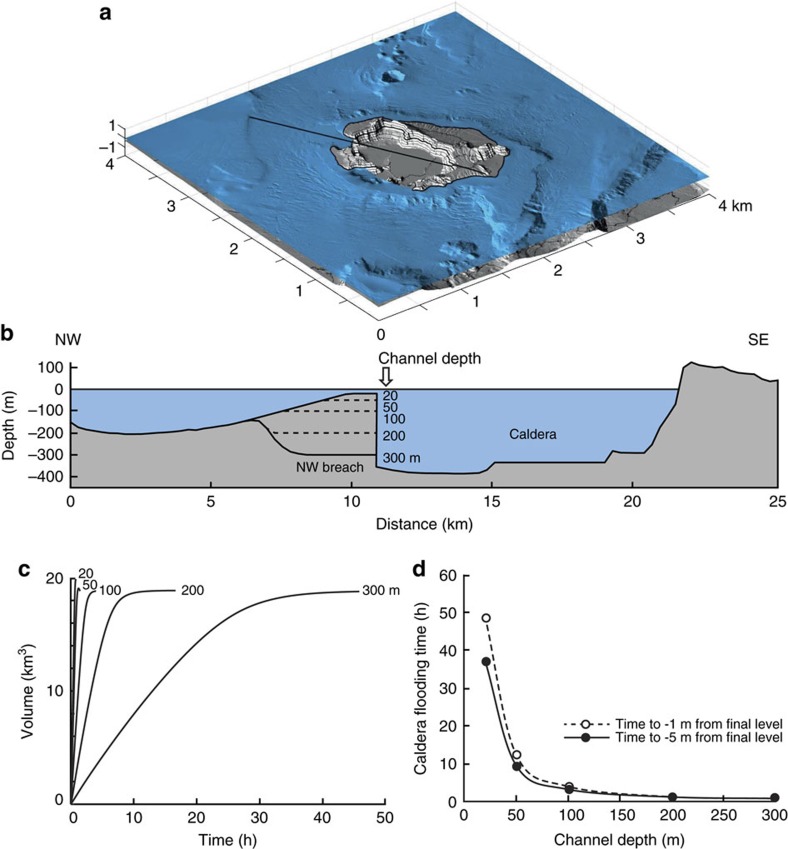
Numerical modeling of flooding. Model set up and results for the flooding of the caldera resulting from the breach of the NW caldera wall. (**a**) Calculation domain for the numerical modelling, showing the initial (pre-flooding) conditions for the 20 m entry model presented in the Supplementary Movie 1. The black line represents the bathymetric profile shown in **b**. (**b**) Bathymetric profile along the traverse in **a**, showing the five different starting profiles with caldera-entry depths of 20, 50, 100, 200 and 300 m. The bathymetry was kept constant over the duration of each model. (**c**) Intracaldera sea water volume as a function of time for the five models. (**d**) Caldera filling time as a function of caldera-entry depth for final conditions of 5 and 1 m below sea level.

The time that it took to fill the caldera was constrained by numerical modelling of the water flow through the NW strait. The model used the depth-averaged equations for water flow and is described in the ‘Methods' section; it neglects shoreline wave breaking, wave energy dispersion and the Coriolis force, but captures the first-order behaviour of water flow in a deep environment^28^. Similar models have been widely used in tsunami modelling^29^. The initial conditions for modelling were created as follows: the caldera floor bathymetry was modified by removing the post-caldera Kameni edifice, which would have been absent immediately following the LBA eruption. An artificial wall was placed across the two SW straits in order to prevent entry of the sea from this direction. The bathymetry of the northern strait was modified by reconstructing the original NW-dipping flank of the volcano using a conical surface with an outward-dipping 2°-slope, then setting the water depth in the breach (equal to the entry depth of the subsequent inundation flow) to a specified value by cutting the cone by a horizontal surface of that depth. In this way we were able to simulate caldera inundation through a series of entry channels of five different specified depths, from 20 to 300 m (Fig. 7). Finally an artificial wall was placed across the NW strait at its entry point into the caldera. This wall was then removed instantaneously in order to allow the sea to flow into the caldera through the NW strait.

For each fixed entry depth, we modelled the inundation of the caldera, and measured the time for filling to −5 m of the final water level, then −1 m of the final water level (Fig. 7c,d). In reality, the entry depth, and indeed the bathymetry of the entire strait, would have evolved with time due to landslip into the caldera, followed by erosion by the rapidly flowing water; however, this time-variation is unknown and cannot be predicted by our model. By fixing the entry depth, and keeping the breach bathymetry constant in each model run, we place constraints on the possible range of caldera-filling times for entry depths >20 m (the lowest value we chose to simulate). The models simply predict caldera-filling times under a range of fixed conditions.

In the 20 m entry depth model, the inflow velocity reached 19 m s^−1^, the water flux reached 2.5 × 10^5^ m^3^ s^−1^, and the filling time was about 50 h. In the 300 m model, the corresponding values were 45 m s^−1^, 92 × 10^5^ m^3^ s^−1^ and 0.6 h. Hence, irrespective of the exact time-variation of breach bathymetry through landslip and water erosion, once the entry depth had reached 20 m, caldera filling would have proceeded to completion in <2 days, and possibly in as little as a few hours. Our model provides no constraints on how long it would have taken to initially erode the entry point to 20 m depth, but given that the NW strait was blocked by unconsolidated LBA tuffs, it seems very likely that, once breached, erosion to this depth would have proceeded very quickly.

Inflow of the sea through the NW strait could have generated large waves inside the caldera (with run-ups up to ∼200 m above the caldera floor on the eastern and southern cliffs), but no significant (amplitude <∼10 m) waves outside the caldera (Supplementary Movie 1).

## Discussion

Tsunamis generated by eruptions at ocean islands are a major hazard worldwide^30^. Those from Krakatau in 1883 impacted the coasts of the Sunda Straits, where run-ups averaged 15 m and reached 40 m, killing 35,000 people^31^,^32^,^33^,^34^,^35^. Tsunamis from the LBA eruption have been proposed as a factor in the demise of the Minoan culture in the southern Aegean region through damage to coastal towns, harbours, shipping and maritime trade (please see refs 36, 37, 38 and references there in). Evidence for regional tsunamis generated by the LBA eruption has been reported from deep sea megaturbidites^39^,^40^,^41^,^42^,^43^,^44^,^45^,^46^ and from sediment layers at or near the coasts of Santorini, northern Crete, west Turkey and Israel^37^,^47^,^48^,^49^,^50^. While some of the sedimentary evidence has been questioned^51^, chaotically deposited debris layers at the Minoan archaeological site of Paleokastro provide particularly convincing evidence for a run-up of at least 9 m along the northeast coast of Crete by tsunamis generated by the LBA eruption^52^.

Tsunamis associated with large explosive eruptions in marine settings are generated by rapid displacement (upward or downward) of the sea surface, the possible mechanisms including submarine explosions, entry into the sea of pyroclastic surges/flows or debris flows, submarine landslides and caldera collapse^30^. A major challenge is the development of reliable forward models with which to predict impacts from such tsunamis. Although existing models are physically robust^29^,^38^,^53^, the relative importance of different tsunami source mechanisms are commonly poorly constrained. In the case of the LBA eruption, modelling of either pyroclastic flow entry into the sea, or caldera collapse, can explain waves of several m on N Crete, depending on exact initial conditions and rates^38^,^48^,^53^. Assuming a pyroclastic flow source, inverse modelling of a 9 m high wave at Palaeokastro implies a wave up to +35 m high, or −11 m deep, at source^52^. This may be an overestimate, since shoreline run-up can overestimate deep-water wave height by a factor of 2 or more due to effects of shoreline configuration, substrate roughness and of wave diffraction, resonance and edge effects^29^. However, even half the inferred initial values are consistent with the occurrence of a sediment layer interpreted as a tsunami deposit on Santorini 10–12 m above sea level^37^.

Submarine explosions during the LBA eruption were mainly confined inside the caldera during the phreatomagmatic phases 2 and 3, and probably radiated little energy outside the caldera. Dense pyroclastic flows of LBA phases 3 and 4 entered the sea in all directions, providing a viable source for major tsunamis^1^,^2^,^3^,^4^,^5^. Indeed pyroclastic flow deposits up to 60-m thick lie offshore Santorini, implying discharge of large volumes of pyroclastic flows into the sea at the peak of the eruption^54^. Multiple thick megaturbidites with volumes of at least 16 km^3^ and containing LBA tephra occur in the Cretan basin to the south of Santorini, and may record large-scale remobilisation of submarine eruption products during and following the eruption^13^.

Our new data on the origin of the NW caldera strait bears on the importance of caldera collapse in tsunami genesis. Caldera collapse associated with the eruption amounted to several hundreds of metres of vertical displacement, and could potentially have generated large tsunamis if it occurred rapidly enough^38^,^53^. However, this requires that the caldera was already flooded and connected to the open sea during collapse, which we have shown was not the case. Although the pre-LBA caldera was lagoonal, it became isolated from the sea and dried up before eruptive phase 4. Caldera collapse during (perhaps continuing after) phase 4 then deepened and widened the old caldera, forming the present-day LBA caldera. Reconnection to the sea then did not take place until the new caldera was flooded through the NW strait after the eruption had ended. It is, moreover, unlikely that the flood event itself could have generated major waves outside the caldera (Supplementary Movie 1). Mass slumping associated with the opening of the NW strait, as well as the later SW straits, would also have generated waves inside the caldera, but would have contributed little to tsunamis on a regional scale.

We conclude that regional-scale tsunamis associated with the LBA eruption were generated by the pyroclastic flow inundation of eruption phases 3 and 4, augmented perhaps by mass slumping of rapidly deposited pyroclastic deposits off the seaward slopes of the island volcano. This is consistent with tsunami modelling that shows that pyroclastic flows were indeed capable of generating waves of the observed height in northern Crete^38^,^53^. It is also consistent with previous assertions that pyroclastic flows were the main cause of tsunamis at Krakatau^31^,^33^,^34^.

## Methods

### Multi-beam surveys

The seabed morphology of Santorini Volcano was investigated by multi-beam surveys by the R/V AEGAEO of the Hellenic Centre for Marine Research (HCMR), using a SEABEAM 2120 swath system as part of international projects ‘GEOWARN' and ‘THERA 2006' (refs 7, 8, 9, 54 and as part of a documentary production ‘ATLANTIS FOUND' in May 2015 using a Teledyne RESON SeaBat7125 MBES system that was mainly operated at 400 Khz. The data were processed using a MB-System for statistical and manual flagging of erroneous beams, and were gridded at 10 and 5 m grid spacing using GMT (the Generic Mapping Tools)^55^.

### Multi-channel seismic surveys

Multi-channel seismic data were collected during RV POSEIDON cruise P338 in 2006 (ref. 56 and during the ‘ATLANTIS FOUND' project in 2015. The respective sources for the high-resolution seismic profiling system were a 45/105. GI-airgun system (SERCEL US) with a dominant frequency of 100 Hz, and a Delta Sparker with frequencies up to 500 Hz. The processing flow included editing, frequency filtering, trace balancing and amplitude loss compensation. Data were edited, bandpass-filtered, CMP-sorted, nmo-corrected, stacked and time-migrated. A poststack f-x deconvolution reduced incoherent noise.

### Digital elevation model

The multi-beam bathymetry point cloud was merged with LIDAR data covering the Kameni Islands and elevation data from Thera and Theresia^9^ and gridded at 10 and 5 m spacings using a continuous curvature algorithm^55^. In the 10 m grid data gaps are interpolated (Figs 1a and 2a). In the 5 m grid data gaps are masked (Fig. 1b–d). Morphological parameters (slope gradient, tangential curvature and flowline density) were computed from the 5 m grid using the /r.slope.aspect/ and /r.flow/ tools from GRASS GIS^57^. The tangential curvature is the curvature of the surface in the direction perpendicular to the maximum slope gradient at each grid point. The flowline density is the density of flowlines per grid cell, where flowlines are particle trajectories calculated by modelling the preferred path of particles starting at each grid cell and moving down slope under the effect of gravity^58^.

### Estimation of eroded volumes

The volume of material eroded from the breach was estimated using a modified version of the GMT resurfacing technique developed for lava flows^9^, with the reconstruction of the pre-erosion surface done in two phases in order to recreate the steep slope inside the caldera (Supplementary Figs 5 and 6). The perspective view of the NW breach (Fig. 2b) was generated from the 10 m grid using Fledermaus.

### Numerical modelling

Numerical modelling of caldera flooding was carried out using the classic shallow water equations of mass (equation 1) and momentum (equations 2 and 3) balances^29^,^59^,^60^,^61^,^62^.


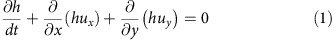



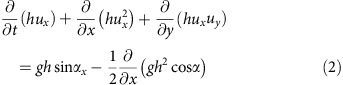



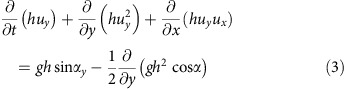


where, *α* is the slope of the sea floor, *u* is velocity, g is gravity and *h* is water depth. The viscosity of water has no influence on the simulation, and is neglected. The terms on the right-hand side of the momentum-balance equations describe the effects of gravity and pressure gradients.

We solved the equations numerically using the code *VolcFlow* (developed and tested in ref. 63), which has been used to simulate tsunamis^64^,^65^,^66^,^67^ using the same equations and boundary conditions as here. The code is based on a double-upwind scheme that limits the numerical dissipation of velocity, and allows calculation of wave amplitudes even at large distances from the source. Depth-averaging in *VolcFlow* is carried out perpendicular to the underlying sea floor. To permit free propagation of surface waves, open boundaries were defined at the border of the model domain (Fig. 7) by calculating the water velocity normal to the border, *u*_b,_ from the water thickness *h*:


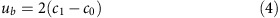


where, 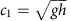 and *c*_0_ equals the value of *c*_1_ at *t*=0. The sea level was maintained constant around the edges of the model domain.

The modelling was carried out using a published digital elevation model of Santorini and its caldera^1^, with the spatial resolution degraded to 125 m (Fig. 7a). The models were run at a spatial resolution of 125 m and a time step of 0.5 s. Changing either parameter did not significantly change the results. Owing to the 125 m discretization of the digital elevation model, the model does not ‘see' a vertical cliff at the entry point of the caldera, but rather a slope ranging from 70° (for the elevation of −20 m) to 26° (for the elevation of −300 m). We investigated the effect of this numerical slope on the model results, and found it to be negligible.

### Data availability

Multi-beam and seismic data are available on request from the corresponding author. The simulation of the filling of Santorini caldera by the sea has been done with the code VolcFlow (http://lmv.univ-bpclermont.fr/volcflow/).

## Additional information

**How to cite this article:** Nomikou, P. *et al*. Post-eruptive flooding of Santorini caldera and implications for tsunami generation. *Nat. Commun.* **7**, 13332 doi: 10.1038/ncomms13332 (2016).

**Publisher's note:** Springer Nature remains neutral with regard to jurisdictional claims in published maps and institutional affiliations.

## Supplementary Material

Supplementary Information Supplementary Figures 1-6.

Supplementary Movie 1 Simulation of the filling of the Santorini caldera by the sea for a 50-m-depth breach. Above is a 3D-view of the simulation. The colours indicate the water depths from the initial state (t=0, dry caldera). Note the small amplitude of the waves generated. Format of the time: hh:mm:ss. The yellow line locates the cross section of the bottom movie. The sea water is in blue, the sea floor in grey.
