# Supplementary material for: Post-eruptive flooding of Santorini caldera and implications for tsunami generation
Source: Nat Commun. 2016 Nov 8;7:13332. doi: 10.1038/ncomms13332 (PMC5105177; doi:10.1038/ncomms13332)
Supplement: Supplementary Information — Supplementary Figures 1-6. [file ncomms13332-s1.pdf]

Supplementary Information

Supplementary Figures

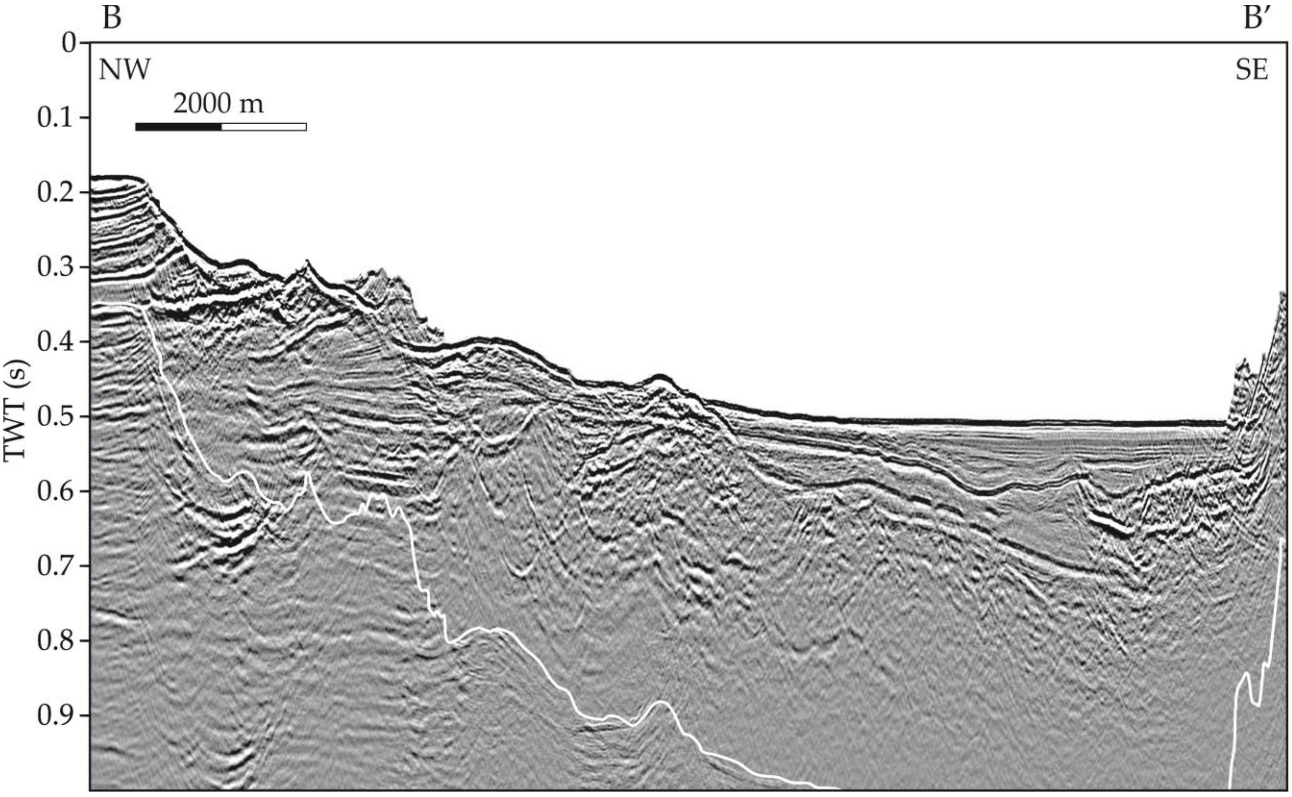

Supplementary Figure 1: Raw un-interpreted multi-channel reflection seismic profile along the axis of the NW strait (see Fig. 3 of the main text).

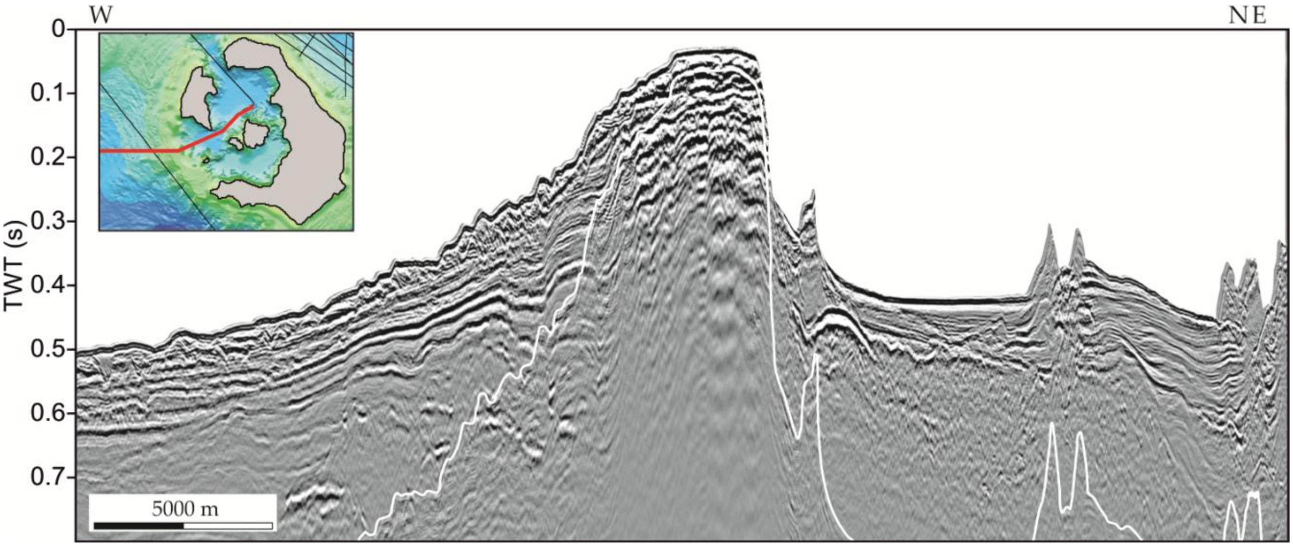

Supplementary Figure 2: Raw un-interpreted W-NE striking multi-channel reflection seismic profile across the SW straits (see Fig. 4 of the main text).

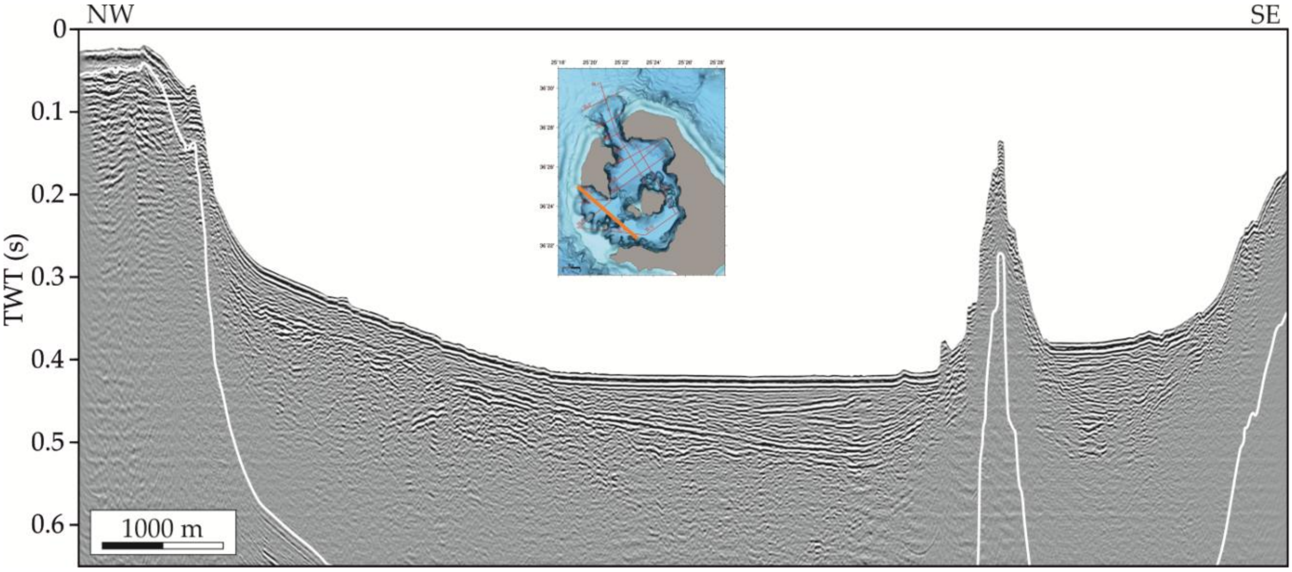

Supplementary Figure 3: Raw un-interpreted NW-SE sparker reflection seismic profile crossing the two SW straits (see Fig. 5 of the main text).

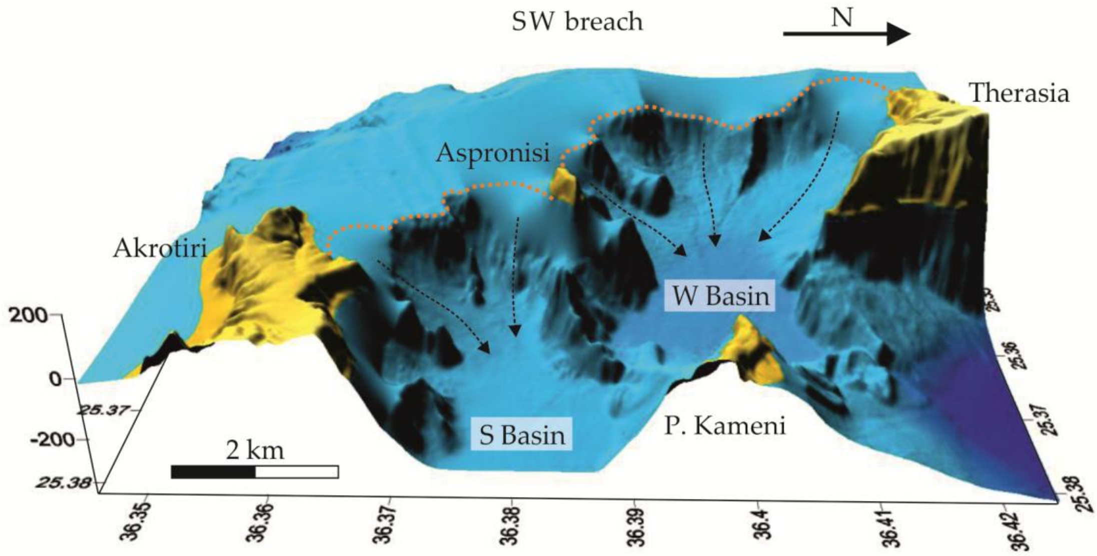

Supplementary Figure 4: 3D topography showing the two SW straits of Santorini caldera between Akrotiri and Therasia. The orange dotted line marks the headwalls of the landslide scars, and the black arrows show schematically the movement directions of those landslides.

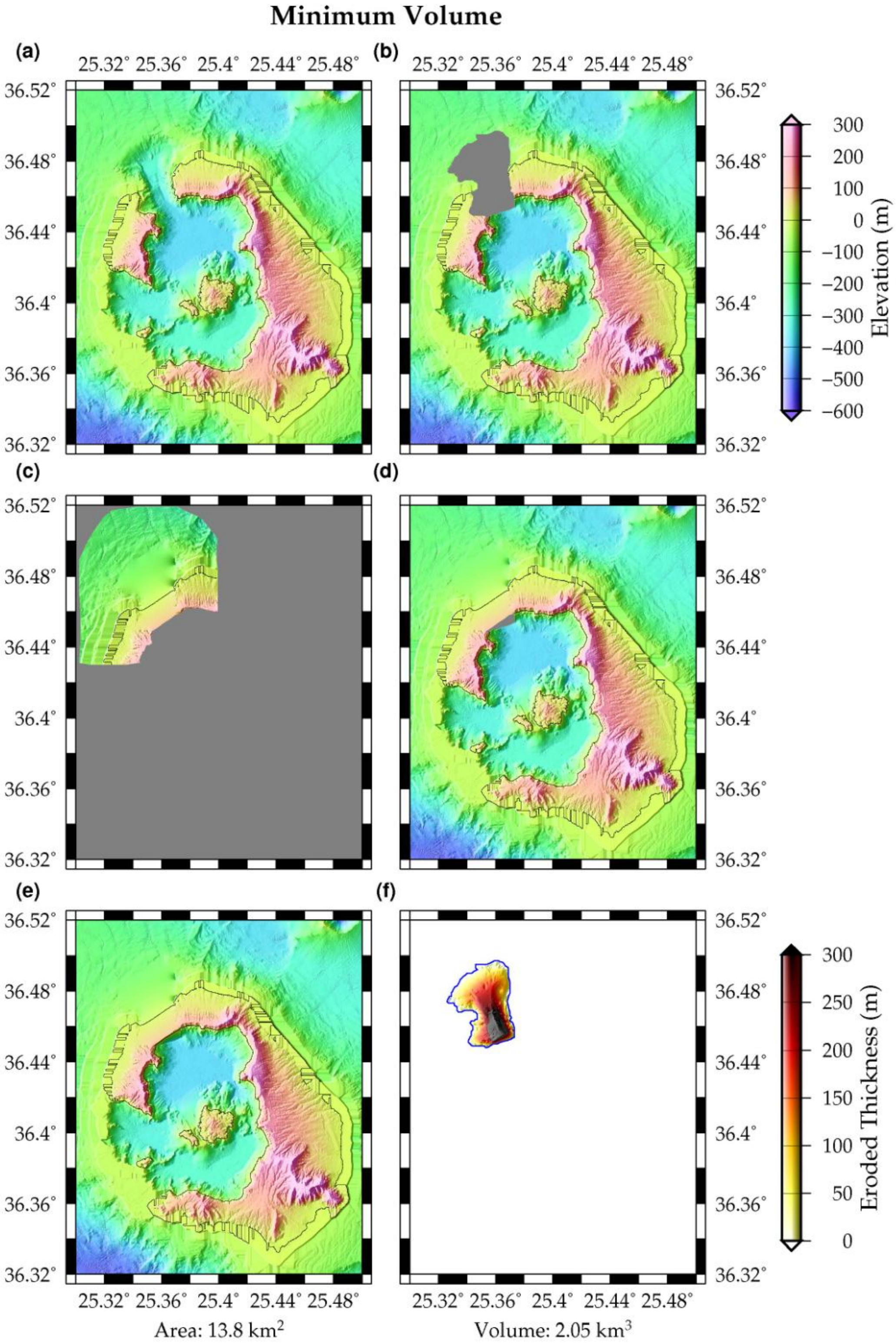

Supplementary Figure 5: A minimum estimate of the volume eroded from the northwest strait. a) Unsmoothed 10 m topography grid. b) Minimum area of breach erosion masked out. c) First step of resurfacing, recreating the gradual outer slope. A limited extent is used to recreate this slope without it being 'pulled down' towards the caldera floor. d) Reconstruction from c) is added into regional bathymetry, leaving a small area masked out on the steep caldera wall. e) A second stage of resurfacing fills in this slope. f) 3D volume of material removed based on the difference between the grids in a) and e), a total volume of 2.05 km^3.

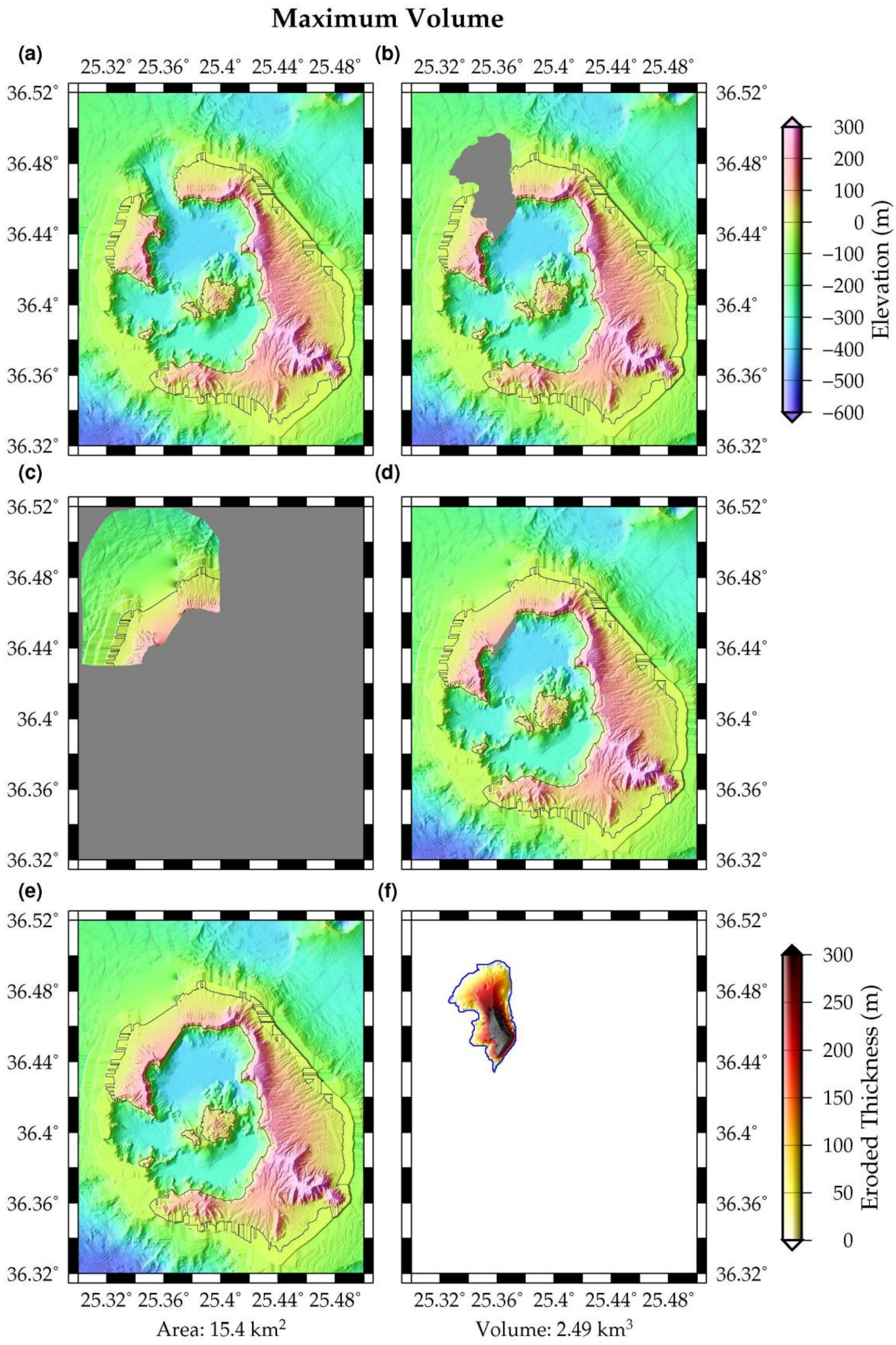

Supplementary Figure 6: A maximum estimate of the volume eroded from the northwest strait. Panels a-f as in Figure 6, but assuming the pre-breach caldera rim was wider, joining the southern of the two small promontories on the western side of the breach, and with a less concave shape. This estimates the maximum amount of material removed, a total volume of 2.49 km^3.
